# Intramuscular fatty infiltration and its correlation with muscle composition and function in hip osteoarthritis

**DOI:** 10.1186/s13395-024-00364-0

**Published:** 2024-12-19

**Authors:** Tatiane Gorski, Nicola C. Casartelli, Gillian Fitzgerald, Astrid M. H. Horstman, Evi Masschelein, Kalliopi J. Essers, Nicola A. Maffiuletti, Reto Sutter, Michael Leunig, Katrien De Bock

**Affiliations:** 1 Laboratory of Exercise and Health, ETH Zurich, Schwerzenbach, Switzerland; 2 Cytometry Facility, University of Zurich, Zurich, Switzerland; 3 Human Performance Lab, Schulthess Clinic, Zurich, Switzerland; 4 Department of Radiology, University Hospital Balgrist, Zurich, Switzerland; 5 Faculty of Medicine, University of Zurich, Zurich, Switzerland; 6 Department of Orthopaedic Surgery, Schulthess Clinic, Zurich, Switzerland; 7 Laboratory of Exercise and Health, Institute of Movement Sciences (D-HEST), ETH Zürich - Swiss Federal Institute of Technology, Schorenstrasse 16, Schwerzenbach, CH-8603 Switzerland

**Keywords:** Intramuscular adipocyte, Knee extension, Total hip replacement, Myofiber, Cross sectional area, Muscle strength, Muscle activation

## Abstract

**Background:**

Hip osteoarthritis patients display higher levels of fatty infiltration (FI) in the gluteus minimus (GM) compared to other hip muscles. We investigated specific histological factors such as fiber type composition and collagen deposition, and functional outcomes like muscle strength and activation associated with FI in these patients.

**Methods:**

In twelve men (67 ± 6 y) undergoing total hip replacement (THR), hip and knee muscle strength and activation (electromyography, EMG) were assessed bilaterally. Magnetic resonance imaging (MRI) was used to compare the relative FI area and muscle cross sectional area (CSA) of the GM, rectus femoris (RF), tensor fascia latae (TFL) and vastus lateralis (VL). Adipocyte content, fiber type composition, grouping, fiber size, centrally nucleated fiber frequency, collagen deposition, satellite cell density and capillarization were assessed in intraoperative biopsies of the four muscles. Differences between GM and other muscles were assessed with repeated-measures one-way ANOVA followed by Dunnett’s post-hoc test. Pearson coefficients were calculated for the correlations between FI measurements and the other histological and functional parameters.

**Results:**

Strength was lower in the affected limb. Knee extensor weakness was accompanied by lower VL muscle activation. Muscle CSA and FI did not differ between sides. In the affected limb, GM displayed larger relative FI area (MRI) compared to RF and VL. Biopsy adipocyte content was higher in GM than RF and TFL. Compared to the other hip muscles, GM displayed higher type 1 fiber content while its type 2X fiber content was lower. Fiber grouping levels were higher in GM than the other muscles. Collagen content was higher in GM than TFL and VL.

FI in GM was associated with type 1 (*r* = 0.43) and type 2X (*r* = -0.34) fiber content, fiber grouping (*r* = 0.39), and collagen deposition (*r* = 0.37). FI in VL was negatively associated with maximal knee extension strength (*r* = -0.65).

**Conclusions:**

In patients undergoing THR, the higher FI levels of GM compared to other hip muscles were associated with fiber type composition and grouping, and with higher collagen deposition. Experimental studies exploring these associations could potentially uncover new targets for the treatment of intramuscular FI and related impairments in muscle function.

**Trial registration:**

KEK number: 2016–01852, date of registration: 12-4-2017.

**Supplementary Information:**

The online version contains supplementary material available at 10.1186/s13395-024-00364-0.

## Introduction

Patients with hip osteoarthritis (HOA) experience hip pain, hip joint stiffness, and hip abductor muscle weakness [1, 2] leading to reduced functional performance, which in turn impairs their quality of life [3] and poses a considerable public health burden [4, 5]. Hip abductor muscle weakness in patients with HOA [1, 2] is often reported without significant differences in muscle size [6] or at least cannot be fully explained by reduced muscle cross-sectional area (CSA) [2]. Such discrepancy between changes in muscle strength and CSA could result from reduced muscle activation and/or loss of muscle quality, the latter including fatty infiltration (FI) [6–9] and/or grouping of myofibers [5]. While fatty infiltration is related to reduced physical capacity, compromised metabolic function in skeletal muscle disorders [7–9], disease severity and poor function in various conditions [10–12], there are currently no pharmacological strategies to counteract or prevent it. In addition, there seems to be variability amongst different muscles, as the gluteal muscles display particularly high levels of FI compared to other hip muscles in patients with HOA [2, 13], but potential differences in quality and function between different muscles around osteoarthritic hips are poorly understood.

Despite several studies on FI in hip skeletal muscles of patients with HOA, little is known about the cellular and microenvironmental factors associated with FI in these patients. In this respect, we recently showed the existence of a subpopulation of fibro-adipogenic progenitors (FAPs) which exhibits a higher adipogenic potential and becomes depleted in the fatty-infiltrated gluteus minimus (GM) muscle [14]. The contribution of other factors within the muscle microenvironment, such as satellite cells (SCs) and blood vessels are associated with HOA, however still needs to be explored. It also remains unclear whether HOA-associated muscle-atrophy results from a reduced myofiber area or whether specific fiber types are more prone to atrophy in HOA. Finally, whether different hip muscles display distinct degrees of re/degeneration and collagen deposition in HOA patients is not known.

In this pilot study, we measured muscle strength, volume, and activation in the hip flexors, hip abductors and knee extensors in both the affected and contralateral side of end-stage HOA patients undergoing total hip replacement (THR). We assessed intramuscular FI levels by means of magnetic resonance imaging (MRI) bilaterally as well as adipose content in biopsies from the GM, tensor fascia latae (TFL), rectus femoris (RF) and vastus lateralis (VL) of the affected side. In addition, we characterized the muscle microenvironment, including myofiber type and size, collagen deposition, capillarization and SC content in these muscle biopsies. We subsequently explored the association between FI and histological and functional properties of these muscles. We hypothesized that FI would be associated with muscle dysfunction, characterized by lower muscle CSA and activation, and higher collagen deposition. These findings will help advancing the understanding of muscle pathology in HOA which in turn can support the design of pre- and rehabilitation programs as well as uncover new (pharmacological) targets for the treatment of FI for end-stage HOA patients undergoing THR.

## Methods

### Patients and study design

Twelve patients undergoing THR for symptomatic HOA were recruited from the Department of Hip Orthopaedic Surgery, Schulthess Clinic (Zürich, Switzerland). Inclusion criteria were end-grade HOA based on clinical and radiological examinations, scheduled for THR, men, and age between 55 and 75 years. Exclusion criteria were OA with Tönnis grade ≥ 2 or pain on the contralateral hip, lower limb surgeries in the previous 10 years, inability to walk without aids, BMI > 35 kg/m^2^, and cardiorespiratory diseases. All patients signed an informed consent before participating in the study. Patient characteristics are described in Table 1. The flow chart of patient recruitment and the study outline are shown in Supplemental Figs. 1 and 2, respectively.

**Table 1:** Patient characteristics

Parameter	Mean ± SD or Number (%)
Age (years)	67 ± 6
**Anthropometrics**	
Body mass (kg)	84 ± 10
Height (cm)	179 ± 4
BMI (kg/m^2^)	26 ± 3
**Hip pain and function**	
Oxford Hip Score (0 to 100, 100 = best score)	50 ± 14
**Physical activity level**	
UCLA current (1 to 10, 10 = highest level)	6 ± 2
**Duration of symptoms (months)**	22 ± 9
**Pain killer intake**	
*Never*	3 (25)
*Sometimes, several times a month*	4 (33)
*Often, several times a week*	2 (17)
*Always, every day*	3 (25)
**Other medication intake**	
*No*	5 (42)
*Yes*	7 (58)

*n*=12

*BMI* body mass index, *UCLA* University of California physical activity scale

The preoperative status of the study participants was evaluated using patient-reported questionnaires. Hip pain and function was evaluated using the Oxford Hip Score [15] and preoperative physical activity level was evaluated using the University of California Los Angeles activity score [16]. Duration of hip symptoms, intake of pain/other medication and presence of problems in other joints were assessed with single questions. Prior to surgery, the degree of FI and CSA of GM, TFL, RF and VL was assessed using MRI and muscle strength and activation (electromyography, EMG) data were collected bilaterally. GM, TFL, RF and VL muscle biopsies were collected for histological analysis during the extracapsular surgical approach for THR. The study was conducted according to the Declaration of Helsinki and the protocol was approved by the Ethics Committee of the Canton of Zurich (KEK number: 2016–01852). Part of the obtained data have been previously reported [14].

### Muscle strength

Isometric maximal voluntary contraction (MVC) strength of the hip abductors [17], hip flexors [18], and knee extensors [19] was assessed bilaterally, using an isokinetic dynamometer (Biodex System 4; Biodex Medical Systems, Shirley, New York). For hip abduction, participants laid on their non-tested side on a treatment table; the tested hip was at 0° of hip flexion, extension, and rotation, with the ipsilateral knee fully extended [17]. The tested hip was abducted to approximately 10°, and the contralateral hip and knee were flexed to 45° and 60°, respectively. The dynamometer rotational axis was aligned with the popliteal fossa of the tested side, and the ipsilateral limb was strapped to the dynamometer pad 5 cm proximal to the medial malleolus. For hip flexion, participants laid supine on the dynamometer chair with the chair back inclined to 15° and the dynamometer rotational axis aligned with the center of hip rotation (greater trochanter) [18]. The tested hip was flexed to 45°, and the ipsilateral thigh was strapped to the dynamometer pad 5 cm proximal to the lateral femoral condyle. For knee extensors, participants sat on the isokinetic dynamometer chair with the chair back inclined at about 90° [19]. The tested knee was fixed at 60° of flexion (0° = knee fully extended). The dynamometer rotational axis was visually aligned to the lateral femoral condyle. The leg was attached to the dynamometer pad 2–3 cm proximal to the lateral malleolus. For each muscle group, participants first performed 5 or 6 submaximal voluntary contractions for familiarization purposes followed by 3 or 4 MVC trials, during which they were asked to maximally contract their muscles for 3–4 s [20]. Rest time between trials was 60 s. Standardized verbal encouragement was consistently provided by the investigators. Additional MVC trials were requested if the difference between the 2 highest MVC torques exceeded 10%. Only the highest MVC torque was retained. After each MVC trial, participants were asked to quantify hip joint pain by placing a vertical mark on a 100-mm horizontal line, known as the visual analog scale. The line ranges from 0 (no pain at all) to 100 (not endurable pain). For each muscle group, the mean pain score was retained.

### Muscle CSA

Muscle CSA of the GM, TFL, RF and VL was evaluated bilaterally by means of non-contrast MRI using the same instrument and protocol adopted for the evaluation of muscle FI [14]. The CSA of the GM, TFL, and RF was assessed on transverse T1-weighted MR images at the level of the upper border of the acetabular rim [13, 21]. In contrast, the CSA of the VL muscle was assessed on transverse T1-weighted MR images at the level of the lesser trochanter. The CSA was quantitatively evaluated by manually drawing contours around the muscle boundaries using the software Picture Archiving and Communication System (PACS, Phoenix Version 5.2 115758, Freiburg, Germany).

### Muscle activity

Muscle activity of the TFL, RF and VL was assessed bilaterally using surface EMG during hip abductor, hip flexion and knee extensor MVC trials, respectively. Note that the GM is not accessible for EMG. Two pairs of silver-chloride surface electrodes (inter-electrode distance of 25 mm) were positioned on the TFL, RF and VL according to standard recommendations [22]. For TFL, electrodes were placed on the line from the anterior spina iliaca superior to the lateral femoral condyle in the proximal 1/6. For RF, electrodes were placed at 50% on the line from the anterior superior iliac spine to the upper border of the patella. And for VL, electrodes were placed at 2/3 on the line from the anterior superior iliac spine to the lateral side of the patella. Low resistance between the two electrodes was achieved with light abrasion of the skin and cleaning with alcohol. The ground electrode was positioned on the ipsilateral patella. EMG signals were amplified with a bandwidth frequency ranging from 10 to 500 Hz (gain 1000), digitized online at a sampling frequency of 2 kHz, and recorded by the Biopac system (MP150, Biopac System Inc., Goleta, CA, USA). Muscle activity was calculated as the EMG root mean square amplitude during 500 ms around MVC using a window length of 125 ms. Only the MVC trial associated to the highest EMG root mean square amplitude was considered [23].

### Muscle fatty infiltration

#### MRI

The degree of FI in the GM, TFL, RF and VL muscles on both the affected and contralateral side was assessed by non-contrast MRI using a 1.5-T system (Avanto-fit, Siemens Healthcare, Erlangen, Germany) according to a standardized protocol. A combination of an 18-channel surface coil and a 32-channel spine coil was used for image acquisition. Coronal T2-weighted fast spin-echo (3990/59 [repetition time ms/echo time ms], echo train length of 15, 4-mm section thickness, 220 × 220-mm field of view, 512 × 512 matrix), sagittal T1-weighted fast spin-echo (641/10, echo train length of 3, 3-mm section thickness, 210 × 210-mm field of view, 320 × 320 matrix), transverse short inversion time inversion-recovery (5550/36/150 [repetition time ms/echo time ms/inversion time ms], echo train length of 13, 7-mm section thickness, 180 × 180-mm field of view, 320 × 320 matrix), transverse T1-weighted fast spin-echo (434/10, echo train length of 2, 6-mm section thickness, 200 × 200-mm field of view, 512 × 512 matrix), and transverse Dixon (6.7/2.4, 5-mm section thickness, echo train length of 2, 200 × 200-mm field of view, 160 × 160 matrix) magnetic resonance (MR) images were obtained. For quantitative assessment of FI, fat fraction maps were generated from the Dixon sequence [24]. For both qualitative and quantitative assessments the GM muscle was assessed on transverse T1-weighted MR images and the fat maps, respectively, at the level of the upper border of the acetabular rim [21], the TFL and RF muscles at the level of femoral head center [25], and the VL muscle at the level of the lesser trochanter. The degree of FI was evaluated quantitatively with the fat fraction maps (0–100%). We used the muscle average of the whole muscle FI for the relation with the functional data (muscle strength, CSA, and activation) and comparison between affected and contralateral limb. Since it is known that FI in the GM varies between different muscle regions [21, 26–30], we additionally assessed FI specifically at the site of the GM biopsy, either at the anterior or mid part of the GM. Correlations between FI and histological outcome parameters were performed on those GM MRI data (Fig. 7a in Fitzgerald et al. [14]). The reproducibility for fatty infiltration of muscle with the Dixon sequence has been assessed, and is highly reliable, with excellent intra- and interobserver reliability (ICC value 0.92, and 0.89, respectively) [31].

#### Biopsy

See below at Histology – Adipose content.

### Muscle biopsy collection

GM, TFL, RF and VL biopsies were collected from the affected side by the same hip surgeon during the extracapsular part of the direct anterior approach used to perform THR. This surgical procedure exposes the four evaluated muscles. For the GM, muscle biopsies were collected from its anterior (*n* = 5) or mid (*n* = 7) part, where the MRI showed the highest FI. A 200 to 500-mg biopsy was collected from each muscle using a scalpel and rapidly embedded in optimal cutting temperature (OCT) medium, frozen in liquid nitrogen-cooled isopentane and stored at -80°C. Transversal 10-µm cryosections of the samples were prepared at -20°C, air dried for ~ 30min and stored at -80°C until further processing. For adipocyte content analysis, samples were cut at -26°C to preserve adipocyte morphology.

### Histology

General tissue morphology, intramuscular adipocyte deposition and frequency of centrally nucleated myofibers were evaluated using haematoxylin and eosin (H&E) staining. Picrosirius red staining was performed as previously described [32] to assess collagen deposition. Immunofluorescence for the different myosin heavy chain (MyHC) isoforms was performed as described elsewhere [33, 34] to assess fiber type composition, size and grouping. SCs and capillaries were detected by immunofluorescence for PAX7 and CD31, respectively, as described previously [35]. The list of antibodies is provided in Table 2. Nuclei were visualized with Hoechst (Thermo Fisher) and samples were imaged with an AxioObserver Z1 fluorescence microscope (Zeiss) using a 10 × objective. H&E-stained samples were imaged with an Eclipse Ti2 inverted microscope (Nikon) using a 10 × objective.

**Table 2:** Antibody information

Antibody	Source	Code	Dilution
MyHC1	DSHB	BA-F8	1:50
MyHC2A	DSHB	SC-71	1:200
MyHC2X	DSHB	6H1	1:100
Laminin	Thermo Fisher	PA1-16730	1:250
Anti-mouse IgG2B Alexa Fluor 488	Thermo Fisher	A-21141	1:250
Anti-mouse IgG1 Alexa Fluor 350	Thermo Fisher	A-21120	1:250
Anti-mouse IgM Alexa Fluor 568	Thermo Fisher	A-21043	1:250
Anti-rabbit IgG Alexa Fluor 647	Thermo Fisher	A-21244	1:250
Anti-PAX7	DSHB	PAX7, supernatant	1:3
Anti-mouse IgG Alexa Fluor 488	Thermo Fisher	A11001	1:250
Anti-human CD31	Dako	M082329-2	1:200

*MyHC* myosin heavy chain

#### Adipocyte content

Adipocyte content was quantified through manually contouring the areas occupied by adipocytes and the total biopsy areas using ImageJ software [36].

#### Fiber type distribution, size and grouping

Fiber type distribution (percentage of total fiber number) and size (minimum Feret’s diameter) were quantified in 287 ± 124 (mean ± standard deviation [SD]) myofibers per sample using Myovision Basic [37]. Since hybrid-type myofibers were absent or present at very low numbers in most biopsies, we excluded them from further analysis. Type 1, type 2A and type 2X grouped fibers were identified as proposed by Kelly et al. [38] for the identification of type 1 fiber groups. Areas of grouped fibers and total fiber area of the biopsies were manually contoured using ZEN 2012 (blue edition).

#### Collagen content and centrally-nucleated fibers

Collagen content was estimated with semi-automatic detection of the Picrosirius red-positive area and centrally-nucleated fibers were manually counted using ImageJ software [36].

#### Capillary parameters

Capillary contacts, individual capillary-to-fiber ratio (C:Fi) and capillary-to-fiber perimeter exchange (CFPE) index of type 1 and remaining (type 2) fibers were determined as described in the literature [39] in four random fields of each biopsy and manually counted using ImageJ [36].

#### Satellite cells (SC)

Pax7-positive nuclei were manually counted as SCs in four random fields of each biopsy using ZEN 2012 (blue edition) software.

### Hip pain, hip function and physical activity level

Hip pain and function were assessed with self-reported questionnaires, i.e. the Oxford Hip Score (OHS) and the hip-oriented Core Outcome Measures Index (COMI-Hip). The OHS is a questionnaire about pain and function. It consists of 12 questions with 5 possible answers [15]. Each question is scored from 0 to 4, where 0 indicates the highest impairment. The scores are added to produce a single score that ranges from 48 to 0, where 0 indicates the highest impairment. The COMI-Hip is a short questionnaire about pain, function, symptom-specific well-being, quality of life, and disability [40]. It consists of 6 questions with 5 possible answers. Each question is scored from 0 to 10, where 10 indicates the highest impairment. The scores are added to produce a single score that ranges from 0 to 10, where 10 indicates the highest impairment. 

The actual level of physical activity—as well as the physical activity level before the occurrence of hip symptoms—was assessed by means of the University of California Los Angeles (UCLA) activity scale [16]. The scale ranges from 1 to 10, with 10 indicating sport participation at the highest intensity.

### Statistics

This was a pilot study aimed at exploring associations between muscle FI and underlying molecular events as well as functional outcomes in humans. Given the lack of (histological) data to use as a basis for a sample size calculation, we considered a sample size of 12 as appropriate [41]. Continuous data are presented as mean ± SD, and ordinal data as median ± range. FI area values obtained with MRI, adipocyte content and frequency of centrally nucleated fibers in the biopsy were log-transformed before further analysis; the transformation resulted in normal distribution of the data, allowing for the use of parametrical tests [42]. Continuous data were tested for normality using D’Agostino-Pearson omnibus normality test, except for type 2X fiber size, for which the sample size in GM was too low and the Shapiro–Wilk test was used. Unless otherwise stated, differences between GM and other muscles were assessed with repeated-measures one-way ANOVA followed by Dunnett’s post-hoc test. The differences in type 2A fiber and type 2X fiber content between GM and other muscles were assessed with Friedman tests followed by Dunn’s post-hoc test since these variables were not normally distributed. Since some biopsies did not contain all fiber types, the differences in fiber size were assessed by fitting a mixed model, followed by a Dunnett’s post-hoc to assess differences between GM and other muscles for each fiber type or a Tukey’s post-hoc test to assess differences between fiber types within each muscle. Normality of the residuals for all parametric tests was assessed with QQ plots. Analyses were conducted using GraphPad Prism 8.2.0. Associations between skeletal muscle fatty infiltrated relative area assessed by MRI or biopsy adipocyte content and other muscle parameters were evaluated using repeated-measures correlations calculated with the rmcorr package in RStudio 1.2.5042. Paired t-tests were used to compare muscle strength, activation and CSA between the affected and contralateral side and Pearson coefficients were calculated for the correlation between FI and strength. Significance level was set at *p* < 0.05.

## Results

Twelve men (age: 67.2 ± 6.1 y, BMI: 26.2 ± 3.4 kg/m^2^) with symptomatic hip disease for 6–30 months participated in this study. Their COMI Hip score was 6.4 ± 1.3, OHS score was 49.8 ± 11.6 and UCLA activity scale changed from 8.2 ± 0.9 before onset of symptoms to 5.7 ± 1.8 at the moment of the study.

### Muscle strength

**Fig. 1:**
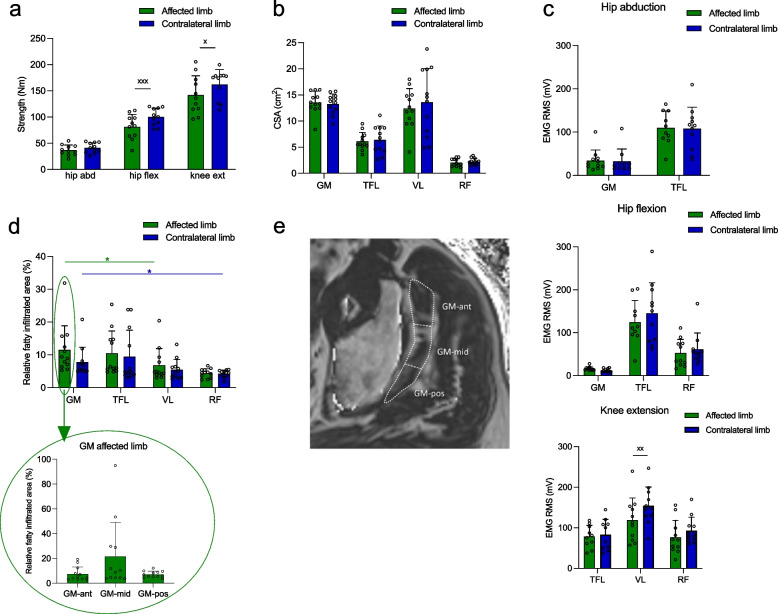
Muscle strength, CSA, activation (EMG) and FI of GM, TFL, VL and RF in the affected and contralateral limb of HOA patients. **a** Strength of the hip abductor, hip flexor and knee extensor muscles. **b** CSA of the GM, TFL, VL and RF. **c** EMG during hip abduction, hip flexion and knee extension. **d** Relative fatty infiltrated area on MRI fat fraction maps in GM, TFL, VL and RF in both limbs. **e** Fat fraction map generated from the transverse Dixon MR-sequence of the left hip in a 74-y old patient, indicating the three GM-regions (GM-ant, GM-mid, GM-pos) at the level of the upper border of the acetabular rim: The signal intensity of each pixel is directly proportional to the relative fatty infiltrated area of the muscle. ^x^ *p* < 0.1, ^xx^ *p* < 0.05, ^xxx^ *p* < 0.005 compared to the affected limb. * *p* < 0.05, ** *p* < 0.005, *** *p* < 0.0005 compared to the GM. CSA, cross sectional area; EMG, electromyography; FI, fatty infiltration; GM, gluteus minimus; HOA, hip osteoarthritis; RF, rectus femoris; TFL, tensor fascia latae; VL, vastus lateralis;

One patient did not do the strength and EMG assessments due to COVID-19. Hip abductor strength did not differ between the affected (36.9 ± 7.9 Nm) and contralateral (41.2 ± 8.3 Nm) side (*p* = 0.251), whereas hip flexion strength was lower on the affected (81.3 ± 18.7 Nm) vs the contralateral (100.3 ± 13.7 Nm) side (*p* = 0.003) and knee extension strength tended to be lower on the affected (142.1 ± 30.0 Nm) vs the contralateral (162.1 ± 23.6 Nm) side (*p* = 0.090) (Fig. 1A).

### Muscle CSA

CSA data from the GM, TFL, VL and RF for both the affected and contralateral sides are shown in Fig. 1B. No significant differences were found between sides (*p* > 0.05).

### Muscle activity

The activity of hip abductor muscles during maximal hip abduction did not differ between the affected and contralateral side (GM: *p* = 0.864, TFL: *p* = 0.807), nor did the activity of hip flexor muscles during hip flexion (RF: *p* = 0.489, TFL: *p* = 0.447) (Fig. 1C). For knee extensor muscles (during maximal knee extension), RF and TFL activity did not differ between sides (RF: *p* = 0.171, TFL: *p* = 0.509), whereas VL activity was significantly lower on the affected vs the contralateral side (*p* = 0.046) (Fig. 1C).

### Muscle FI

#### MRI

The relative fatty-infiltrated area was not different between the affected and contralateral sides for any of the muscles (*p* > 0.05, Fig. 1D).

Within both the affected and the contralateral limb, FI in GM was higher than in RF (*p* = 0.011 and *p* = 0.017, resp.), whereas there was no difference between GM and TFL, nor between GM and VL (p > 0.05) (Fig. 1D). In the affected limb, the relative fatty infiltrated area did not significantly differ between the anterior, mid and posterior part of the GM (Fig. 1D lower part and E).

#### Biopsies

**Fig. 2:**
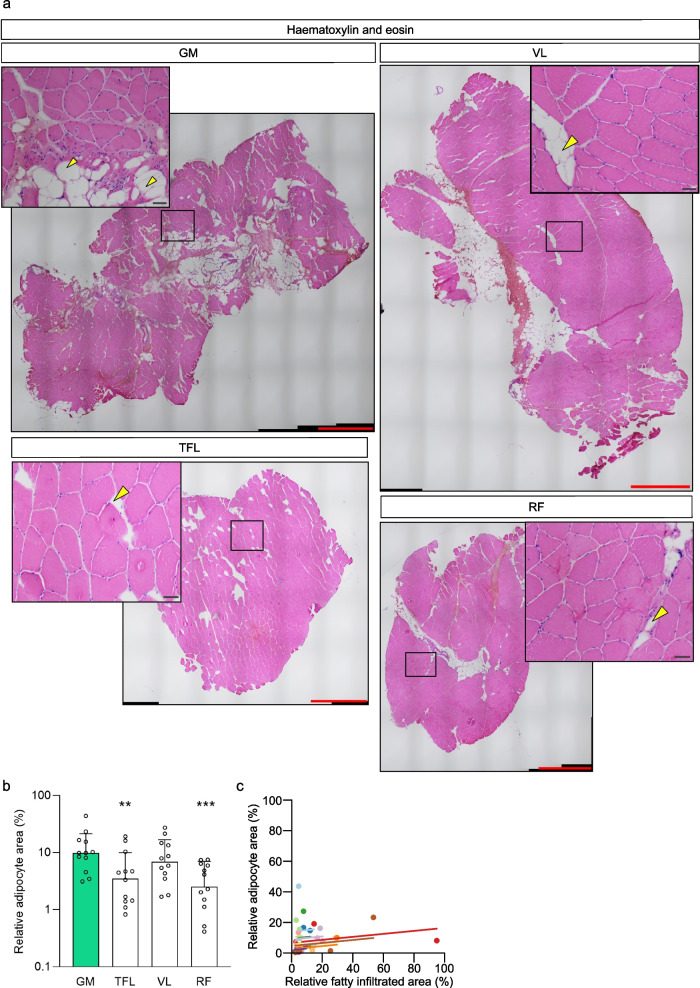
Fatty infiltration of GM, RF, TFL and VL in affected limb of HOA patients. **a** Representative images of haematoxylin and eosin (H&E)-stained sections. Yellow arrowheads indicate intramuscular adipocytes. Scale bars, 1000 µm; scale bars in the inserts, 50 µm. **b** Relative adipocyte area in the biopsies. **c** Repeated-measures correlation between the relative fatty infiltrated area assessed with MRI and the relative adipocyte content assessed with H&E staining in the skeletal muscle biopsies. Values from each patient are displayed in one color, with corresponding lines to show the repeated-measures correlation fit for each patient. Data presented as mean and standard deviation (**b**). Data were analyzed using repeated-measures one-way ANOVA and Dunnett’s post-hoc test (**b**), or repeated-measures correlation (**c**). * *p* < 0.05, ** *p* < 0.005, *** *p* < 0.0005 compared to the GM. GM, gluteus minimus; HOA, hip osteoarthritis; RF, rectus femoris; TFL, tensor fascia latae; VL vastus lateralis

The adipocyte content observed in the biopsies was higher in GM compared to RF and TFL (*p* = 0.0007, *p* = 0.0097, resp.) and similar to VL (*p* = 0.571) (Fig. 2A, B). When all muscles were pooled for repeated-measures correlation, there was no correlation between relative FI area assessed with MRI and adipocyte content assessed on histological sections (*r* = 0.176, *p* = 0.297, Fig. 2C). A negative correlation was found between knee extension strength and FI in the knee extensor muscle VL (biopsy) (*r* = -0.651, *p* = 0.030).

### Histology

#### Fiber type distribution, size and grouping

**Fig. 3:**
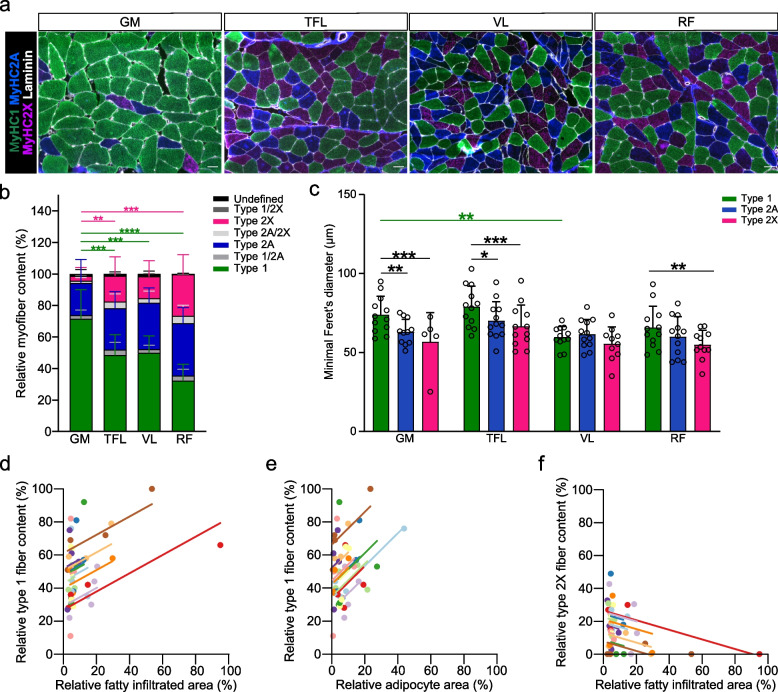
Fiber type composition of GM, TFL, VL and RF in affected limb of HOA patients. **a** Representative images of fiber type immunofluorescence. Laminin staining is displayed in white, MyHC1 staining in green (type 1), MyHC2A (type 2a) in dark blue and MyHC2X (type 2b) in violet. Scale bars, 50 µm. **b** Relative fiber type content assessed in the muscle biopsies. **c** Fiber size according to type. **d–f** Repeated-measures correlations between Type 1 fiber content and relative fatty infiltrated area assessed by magnetic resonance imaging (MRI) (**d**) or adipocyte area in the biopsies (**e**), and between type 2X fiber content and relative fatty infiltrated area assessed by MRI (**f**). Values from each patient are displayed in one color, with corresponding lines to show the repeated-measures correlation fit for each patient. **b**, **c** Data presented as mean and standard deviation. Data were analyzed using repeated-measures one-way ANOVA and Dunnett’s post-hoc test (**b**, type 1 fibers), Friedman tests and Dunn’s post-hoc (**b**, type 2A and 2X fibers), mixed model followed by Dunnett’s post-hoc to assess differences between the GM and other muscles, or by Tukey’s post-hoc to assess differences between fiber types (**c**) or repeated-measures correlation (**d**-**f**). * *p* < 0.05, ** *p* < 0.005, *** *p* < 0.0005, **** *p* < 0.0001 compared to the GM. GM, gluteus minimus; HOA, hip osteoarthritis; RF, rectus femoris; TFL, tensor fascia latae; VL, vastus lateralis

Fiber type compositions of all four muscles on the affected side are shown in Fig. 3A. GM displayed higher type 1 fiber content when compared to all other muscles (Fig. 3B). Also, GM had the lowest type 2X fiber content (Fig. 3B). In GM and TFL, Type 1 fibers were larger than both type 2A and type 2X fibers (Fig. 3C). In RF, type 1 fibers were larger than type 2X, but not type 2B fibers. There were no differences in fiber diameter between muscle fiber types in VL. Type 1 fibers from the GM were larger than those of the VL muscle (*p* = 0.003), but not significantly different from RF or TFL (*p* = 0.136 and 0.472, resp.). The diameter of type 2A and type 2X fibers did not differ between any of the muscles (*p* > 0.05).

Including all muscle samples, we observed a correlation between the relative content of type 1 fibers and the degree of FI assessed with MRI (*r* = 0.438, *p* = 0.007, Fig. 3D) or biopsy adipocyte content (*r* = 0.434, *p* = 0.007, Fig. 3E), as well as a negative correlation between type 2X fiber content and the FI degree assessed with MRI (*r* = -0.344, *p* = 0.037, Fig. 3F).

**Fig. 4:**
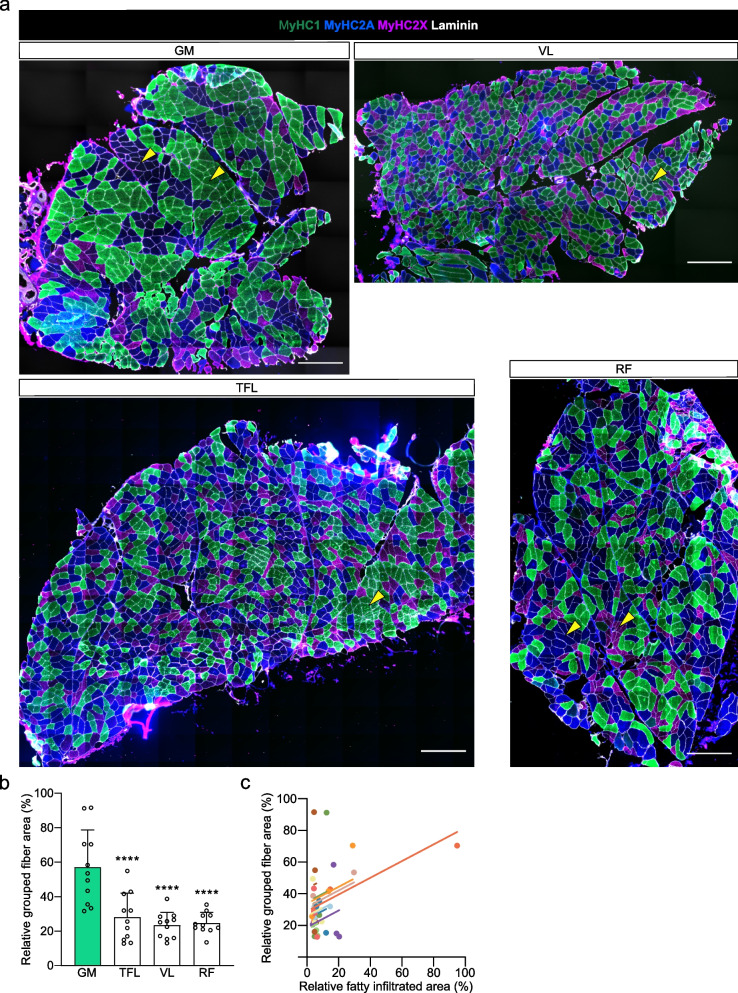
Fiber type grouping levels of GM, TFL, VL and RF in affected limb of HOA patients. **a** Representative images of fiber type grouping. Laminin staining is displayed in white, MyHC1 staining in green, MyHC2A in dark blue and MyHC2X in violet. Scale bars, 50 µm. Yellow arrowheads indicate grouped fibers. **b** Relative area occupied by grouped fibers. Data are presented as mean and standard deviation and were analyzed using repeated-measures one-way ANOVA and Dunnett’s post-hoc test. **** *p* < 0.0001 compared to the GM muscle. **c** Repeated-measures correlation between the relative fatty infiltrated area assessed by MRI and the fiber grouping levels. Values from each patient are displayed in one color, with corresponding lines to show the repeated-measures correlation fit for each patient. GM, gluteus minimus; HOA, hip osteoarthritis; RF, rectus femoris; TFL, tensor fascia latae; VL, vastus lateralis

The GM displayed the highest fiber type grouping levels, measured as relative area occupied by grouped fibers in each biopsy, of the four muscles (Fig. 4A, B). Furthermore, fiber grouping levels correlated with relative fatty infiltrated muscle area assessed by MRI (*r* = 0.386, *p* = 0.024, Fig. 4C).

#### Collagen content and centrally-nucleated fibers

**Fig. 5:**
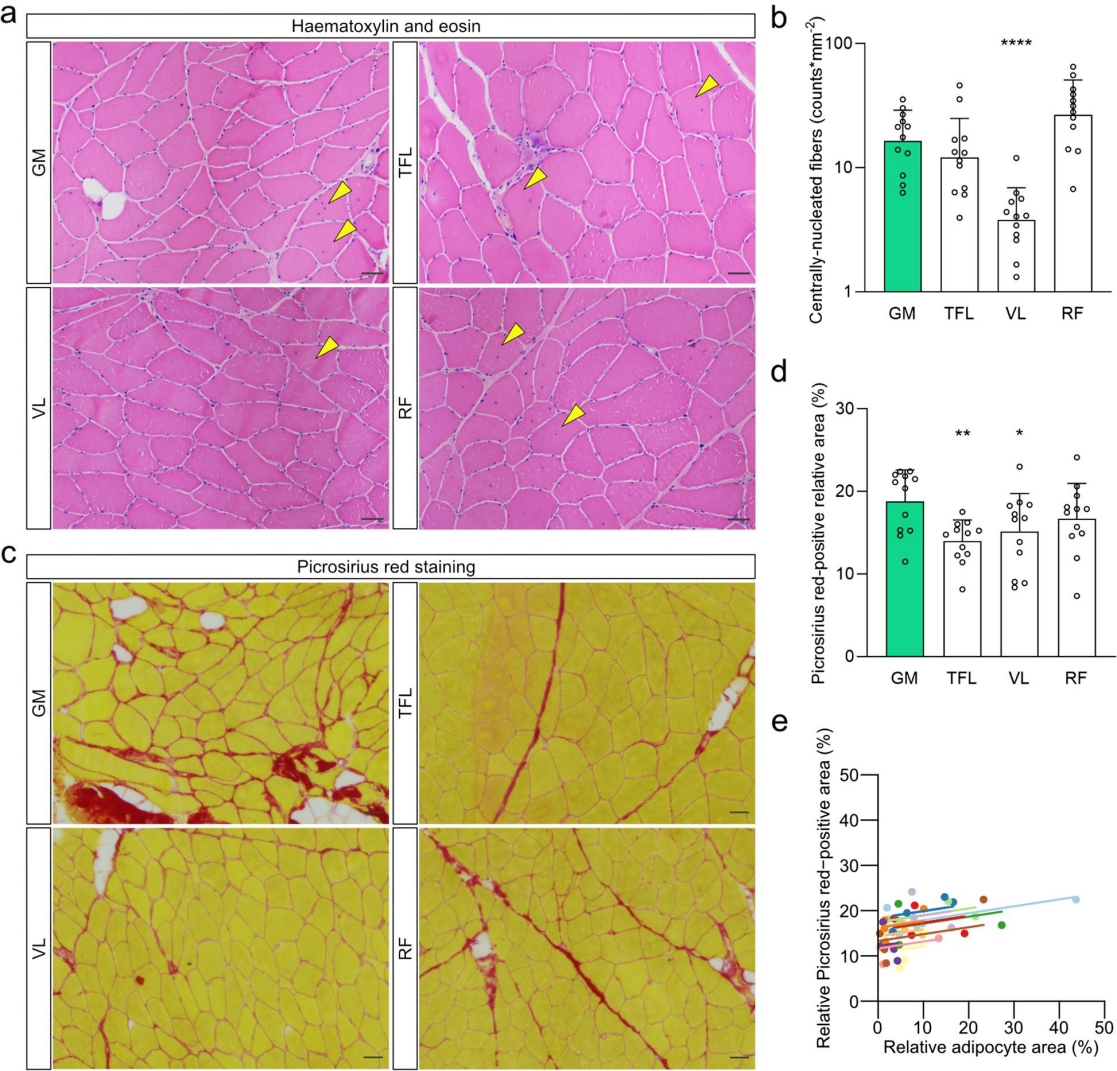
Collagen deposition between GM, TFL, VL and RF fibers in affected limb of HOA patients. **a** Representative images of hematoxylin and eosin staining in cross-sections. Yellow arrowheads indicate centrally nucleated fibers. Scale bars, 50 µm. **b** Frequency of centrally nucleated fibers. Note the logarithmic scale on the y axis. **c** Representative images of Picrosirius red staining. **d** Collagen content evaluated as the Picrosirius red-positive relative area. Scale bars, 50 µm. **e** Repeated-measures correlation between the Picrosirius red-positive relative area and adipocyte area in the skeletal muscle biopsies. Values from each patient are displayed in one color, with corresponding lines to show the repeated-measures correlation fit for each patient. **b**, **d** Data are presented as mean and standard deviation. Data were analyzed using repeated-measures one-way ANOVA and Dunnett’s post-hoc test (**b**, **d**) or repeated-measures correlation (**e**). * *p* < 0.05, ** *p* < 0.005, **** *p* < 0.0001 compared to the GM. GM, gluteus minimus; HOA, hip osteoarthritis; RF, rectus femoris; TFL, tensor fascia latae; VL, vastus lateralis

Centrally-nucleated myofibers were more frequent in the GM than in the VL muscle (*p* < 0.0001), but the other muscles – including the RF, which consistently displayed lower levels of FI in both MRI and biopsy analysis – did not have different numbers of centrally nucleated fibers when compared to the GM (*p* > 0.05, Fig. 5A, B).

Intramuscular collagen deposition was higher in the GM than in the TFL and VL muscles (*p* = 0.001 and 0.016, resp., Fig. 5C, D). In addition, when all samples were pooled for repeated-measures correlation, we observed a correlation between collagen content and adipocyte content in the biopsies (*r* = 0.367, *p* = 0.025, Fig. 5E).

#### Capillary parameters

**Fig. 6:**
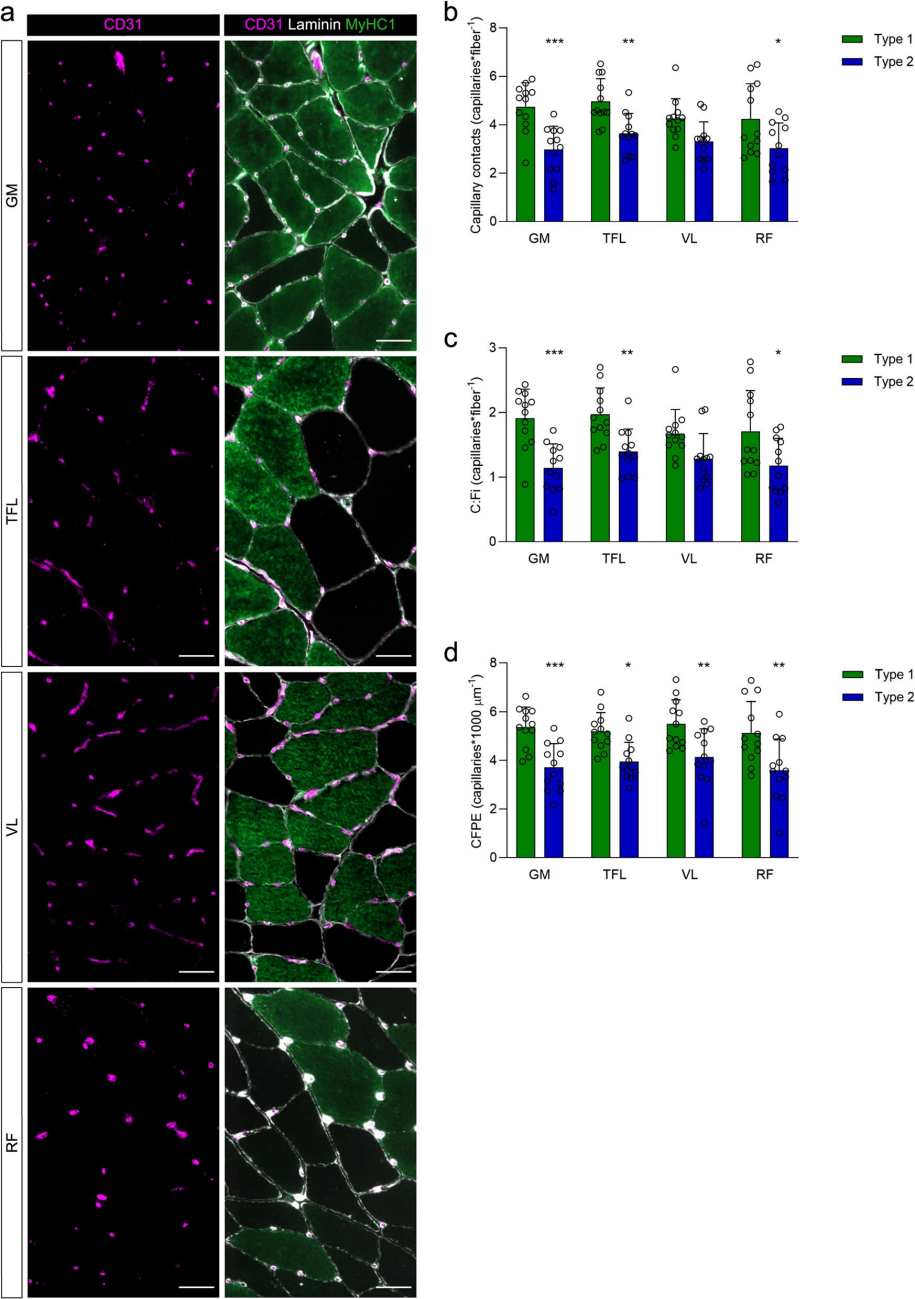
Capillary supply of GM, TFL, VL and RF in affected limb of HOA patients. **a** Representative immunofluorescence images of capillaries stained with CD31 (violet), MyHC 1 (green) and Laminin (white). Scale bars, 50 µm. **b** Capillary contacts per fiber, (**c**) capillary to fiber ratio calculated on an individual basis (C:Fi) and (**d**) capillary-to-fiber perimeter exchange index (CFPE index) of type 1 and type 2 fibers. Data were analyzed using repeated-measures two-way ANOVA and Sidak’s post-hoc test. * *p* < 0.05, ** *p* < 0.005, *** *p* < 0.0005 compared to type 1 fibers within each muscle. GM, gluteus minimus; HOA, hip osteoarthritis; RF, rectus femoris; TFL, tensor fascia latae; VL, vastus lateralis

Type 1 fibers displayed more capillary contacts, as well as higher C:Fi and CFPE compared to type 2 fibers in all muscles, except for the capillary contacts and C:Fi in the VL muscle (Fig. 6). However, these parameters were not different between the GM and the other hip muscles, and did not correlate with the degree of FI assessed by MRI or the adipocyte content in the biopsies.

#### Satellite cells (SC)

**Fig. 7:**
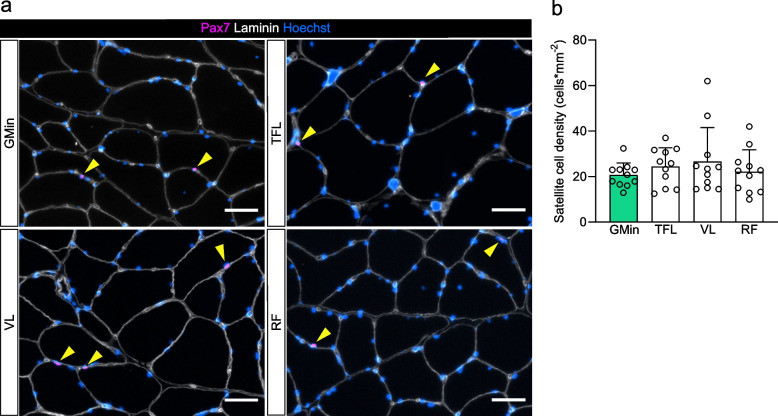
Satellite cell density of GM, TFL, VL and RF in affected limb of HOA patients. **a** Representative immunofluorescence images of Pax7 (violet), Hoechst (blue) and Laminin (white). Yellow arrowheads indicate satellite cells (Hoechst-positive, Pax7-positive nuclei). Scale bars, 50 µm. **b** Satellite cell density. Data are presented as mean and standard deviation and were analyzed using repeated-measures one-way ANOVA and Dunnett’s post-hoc test. GM, gluteus minimus; HOA, hip osteoarthritis; RF, rectus femoris; TFL, tensor fascia latae; VL, vastus lateralis

One patient was excluded from fiber grouping and SC density analysis due to low fiber number in the GM biopsy. The SC density was not different between the GM and the other hip muscles (Fig. 7A, B); accordingly, SC density and FI levels assessed by MRI or in the biopsies did not correlate (*r* = -0.017, *p* = 0.924).

## Discussion

Here, we found that the muscle size and FI levels in the affected and contralateral hips of patients with HOA were similar, and do not explain strength deficits in the affected hip. However, taking advantage of the higher FI levels in the GM compared to other hip skeletal muscles, we explored the microstructural factors associated with this condition in skeletal muscle biopsies from HOA patients. In these patients, FI was associated with a higher type 1 fiber content, which came at the expense of a lower type 2X fiber content, increased fiber type grouping and an expansion of the ECM.

### Muscle strength, CSA and activity

Contrary to earlier findings in a systematic review in which patients with HOA show lower hip muscle strength in the affected limb [43], we did not find differences in hip abductor strength between affected and contralateral side, while knee extension and hip flexion strength were lower in the affected side. This was not caused by lower muscle CSA. While the activity of hip abductor and flexor muscles did not differ between sides, knee extensor VL muscle activity was lower in the affected, as was also found by Suetta et al. [44]. Therefore, deficits in muscle activity seem to, at least partially, contribute to knee extensor muscle weakness in patients with HOA.

### Fatty infiltration

No differences were found in FI between the two sides. However, in line with previous findings [2, 13], we observed distinct levels of FI assessed by MRI between the hip muscles analyzed. Relative FI was higher in the GM compared to RF, and the adipocyte content observed in the biopsies was higher in GM compared to RF and TFL and similar to VL.

FI has been suggested to reduce the availability of contractile, force-generating tissue leading to muscle weakness and reduced function [7]. But similar to previous work [45], we did not find a correlation between FI and hip muscle strength in our patients. However, we did find a negative correlation between FI of the VL and knee extensor strength in patients with HOA, suggesting that also the quality of muscles other than hip muscles are affected in HOA.

### Fiber type distribution, size and grouping

FI was associated with a higher type 1 fiber content and increased fiber type grouping. Our results are in line with previous work estimating a predominance of type 1 fibers, followed by type 2A and type 2X fibers in the GM and a higher amount of type 1 fibers in the GM compared to the VL in patients undergoing THR [46]. In addition, a predominance of type 1 fibers, independent of the FI levels in those muscle regions, has been reported in elderly subjects [26]. This indicates that the high percentage of type 1 fibers observed in the GM compared to other hip muscles may precede HOA or FI. It remains to be experimentally demonstrated whether type 1 fibers promote a more permissive microenvironment for the adipogenic differentiation of progenitor cells residing in skeletal muscle.

Type 1 fibers were larger than type 2A and/or 2X fibers in all muscles analyzed, except the VL. This is similar to previous studies reporting larger sizes for type 1 than type 2 fibers in the GM, TFL and gluteus medius muscles of older adults with or without HOA [26, 47]. This pattern could result from the specific atrophy of type 2 fibers in these muscles, as described during aging [48]. In fact, the atrophy of type 2 fibers is more prevalent than the atrophy of type 1 fibers in the vastus medialis of patients with knee OA [49], and HOA was suggested to aggravate the aging-related selective atrophy of type 2 fibers in the TFL [47]. The VL was the only muscle where size was similar between all fiber types, implying that the atrophy of type 2 fibers was less pronounced in this muscle compared to the other muscles analyzed. The lack of correlation between fiber size and FI indicates that factors other than fiber atrophy may contribute to the development of intramuscular FI in HOA.

Fiber type grouping has been commonly reported in aging human skeletal muscle, likely resulting from the denervation and subsequent reinnervation of myofibers by neighboring motor neurons [50]. Since both grouping of type 1 and/or type 2 fibers as well as FI have been reported in the vastus medialis of patients with knee OA [49] and in hip muscles of patients with HOA [47, 51], we explored whether these two alterations were associated in patients with HOA. We observed significant fiber type grouping levels in the GM compared to the other hip muscles, suggesting higher rates of fiber denervation and remodeling of motor units in this muscle, which may contribute to, or result from, the development of FI.

This higher fiber grouping and the association between fiber type grouping and FI resemble experimental work showing intramuscular adipocyte deposition following skeletal muscle denervation in rabbits [52]. Future experiments may demonstrate whether fiber denervation per se can lead to FAP adipogenic differentiation, and/or whether denervated fibers lose an eventual ability to suppress this process. In fact, healthy myogenic cells/myotubes inhibit FAP adipogenic differentiation in vitro [53]. Alterations in Schwann cell function due to denervation could also lead to FAP adipogenesis, since these cells suppress FAP adipogenic differentiation in a mouse model of chemically-induced regeneration [54]. Motor unit remodeling could also lead to FI by increasing type 1 fiber content, should type 1 fibers promote a more permissive environment for adipogenesis.

The criteria used here to assign fibers as grouped, considering fiber type distribution and the consequent probability of similar adjacent myofibers, were intended to minimize the influence of fiber type distribution on the grouping levels detected [38]. However, the high fiber grouping levels observed in the GM may partially result from a higher type 1 fiber content and lower type 2 fiber content in this muscle compared to the other muscles, since grouping levels correlated significantly with the content of the three fiber types analyzed.

### Collagen content and centrally-nucleated fibers

Intramuscular collagen content was higher in the GM than in the TFL and VL muscles. In addition, we observed a positive correlation between collagen content and adipocyte content in the biopsies. This suggests that intramuscular adipocyte deposition is also associated with other dysfunctional phenotypes of cell populations residing in skeletal muscle. In this respect, in experimental mouse models, adipocyte deposition can result from the dysfunctional differentiation of FAPs into adipocytes in re/degeneration settings [53]. Importantly, there was no correlation between the FI indexes and the number of centrally-nucleated myofibers, indicating that FI does not seem to be a direct result/cause of altered myofiber re-/ or degeneration. Based on this and on the positive correlation between collagen and adipocyte content observed in the biopsies, we speculate that the GM microenvironment could promote aberrant fate decisions of progenitor cells not only towards the adipogenic, but also towards the fibrogenic fate.

### Capillary parameters and satellite cells (SC)

Skeletal muscle SC content is reduced during aging [48] and in the VL of patients with knee OA [55]. In mice, ablation of SCs leads to defective muscle regeneration, and extensive adipocyte deposition [56]. We therefore investigated whether differences in SC content between the GM and other hip muscles could contribute to FI. The SC density was however not different between the GM and the other hip muscles and did not correlate with FI levels.

Since skeletal muscle FAPs tend to reside in close proximity to blood vessels [53], we hypothesized that changes in vascularization could affect FAP function and contribute to the deposition of intramuscular adipocytes in the GM of HOA patients. However, muscle capillarization was not different between the GM and the other hip muscles, and did not correlate with the degree of FI. Therefore, FI seems to occur independently of SC content and capillary supply in hip muscles of HOA patients.

### Clinical relevance

Our findings advance the understanding of muscle pathology in hip osteoarthritis, which in turn can support the design of pre- and rehabilitation programs for end-stage HOA patients undergoing THR as well as the design of future studies that aim to uncover the pathophysiological mechanisms of FI. Indeed, experimental therapies targeting FAPs may offer new ways to manage FI in HOA. The extensive characterization of the skeletal muscle microenvironment associated with FI provides a basis for the development of more precise and effective treatment strategies. By addressing the specific needs of muscles like the GM, which are disproportionately affected by FI, healthcare providers can improve functional outcomes and quality of life for patients with hip osteoarthritis.

### Limitations

The main purpose of this pilot study was to explore the microstructural factors related to FI in different skeletal muscle biopsies from HOA patients. For this reason, no healthy controls were included in the current study, and we were only able to compare the affected to the contralateral limb for the MRI and functional outcome measures. Our conclusions therefore are limited to patients with HOA, and our observations cannot be causally linked to the development of HOA. Since we analyzed intraoperative biopsies of HOA patients undergoing THR, only biopsies taken from the OA-affected side could be obtained and evaluated, preventing us from distinguishing between OA-, aging-, and muscle-specific factors associated with the development of intramuscular FI. Further studies comparing the muscles from the affected to the contralateral side, as well as to healthy controls or in experimental animal studies, may elucidate how fiber type composition and grouping, and increased collagen deposition and SC density relate to intramuscular FI specifically in OA. While our findings may support the design of prehabilitation and rehabilitation programs for end-stage HOA patients undergoing THR, further research with larger sample sizes is needed.

## Conclusions

Our findings shed light on the cellular composition of hip muscles in patients with HOA and how it may relate with intramuscular FI. Besides providing reference values for different skeletal muscle parameters in end-stage HOA patients undergoing THR, our findings suggest that FI differently affects hip muscles, and that the higher FI levels in the GM are associated with higher collagen content, higher type 1 fiber content and fiber grouping. In addition, FI of the VL muscle negatively correlated with knee extensor strength. Future studies experimentally testing the associations observed in this study could uncover new targets for the treatment or prevention of FI in risk groups, such as e.g. exercise, nutritional or pharmacological interventions, counteracting the knee and hip muscle weakness observed in HOA patients.

## Supplementary Information

Supplementary Material 1: Suppl. Fig. 1: Flow chart.

Supplementary Material 2: Suppl. Fig. 2: Study outline.

## Data Availability

The datasets used and/or analyzed during the current study are available from the corresponding author on reasonable request.

## Abbreviations

BMI: Body mass index
CFi: Capillary-to-fiber ratio
CFPE: Capillary-to-fiber perimeter exchange
COMI-Hip: Hip-oriented core outcome measures index
CSA: Cross-sectional area
CT: Computerized tomography
EMG: Electromyography
FAPs: Fibro-adipogenic progenitors
FI: Fatty infiltration
GM: Gluteus minimus muscle
H&E: Haematoxylin and eosin
HOA: Hip osteoarthritis
MR: Magnetic resonance
MRI: Magnetic resonance imaging
MVC: Maximal voluntary contraction
MyHC: Myosin heavy chain
OA: Osteoarthritis
OCT: Optimal cutting temperature
OHS: Oxford Hip Score
PACS: Picture Archiving and Communication System
RF: Rectus femoris
SC: Satellite cell
TFL: Tensor fascia latae
THR: Total hip replacement
UCLA: University of California Los Angeles
VL: Vastus lateralis

## References

[1] Arokoski MH, Arokoski JP, Haara M, Kankaanpaa M, Vesterinen M, Niemitukia LH, et al. Hip muscle strength and muscle cross sectional area in men with and without hip osteoarthritis. J Rheumatol. 2002;29(10):2185–95. 12375331

[2] Rasch A, Byström AH, Dalen N, Berg HE. Reduced muscle radiological density, cross-sectional area, and strength of major hip and knee muscles in 22 patients with hip osteoarthritis. Acta Orthop. 2007;78(4):505–10. 17966005 10.1080/17453670710014158

[3] Salaffi F, Carotti M, Stancati A, Grassi W. Health-related quality of life in older adults with symptomatic hip and knee osteoarthritis: a comparison with matched healthy controls. Aging Clin Exp Res. 2005;17(4):255–63. 16285189 10.1007/BF03324607

[4] Murphy LB, Helmick CG, Schwartz TA, Renner JB, Tudor G, Koch GG, et al. One in four people may develop symptomatic hip osteoarthritis in his or her lifetime. Osteoarthritis Cartilage. 2010;18(11):1372–9. 20713163 10.1016/j.joca.2010.08.005 PMC2998063

[5] Rydevik K, Fernandes L, Nordsletten L, Risberg MA. Functioning and disability in patients with hip osteoarthritis with mild to moderate pain. J Orthop Sports Phys Ther. 2010;40(10):616–24. 20811166 10.2519/jospt.2010.3346

[6] Marshall AR, Noronha M, Zacharias A, Kapakoulakis T, Green R. Structure and function of the abductors in patients with hip osteoarthritis: Systematic review and meta-analysis. J Back Musculoskelet Rehabil. 2016;29(2):191–204. 26406195 10.3233/BMR-150614

[7] Addison O, Marcus RL, Lastayo PC, Ryan AS. Intermuscular fat: a review of the consequences and causes. Int J Endocrinol. 2014;2014:309570. 24527032 10.1155/2014/309570 PMC3910392

[8] Hilton TN, Tuttle LJ, Bohnert KL, Mueller MJ, Sinacore DR. Excessive adipose tissue infiltration in skeletal muscle in individuals with obesity, diabetes mellitus, and peripheral neuropathy: association with performance and function. Phys Ther. 2008;88(11):1336–44. 18801853 10.2522/ptj.20080079 PMC2579904

[9] Goodpaster BH, Thaete FL, Kelley DE. Thigh adipose tissue distribution is associated with insulin resistance in obesity and in type 2 diabetes mellitus. Am J Clin Nutr. 2000;71(4):885–92. 10731493 10.1093/ajcn/71.4.885

[10] Hogarth MW, Uapinyoying P, Mazala DAG, Jaiswal JK. Pathogenic role and therapeutic potential of fibro-adipogenic progenitors in muscle disease. Trends Mol Med. 2022;28(1):8–11. 34750068 10.1016/j.molmed.2021.10.003 PMC11969197

[11] Marcus RL, Addison O, Dibble LE, Foreman KB, Morrell G, Lastayo P. Intramuscular adipose tissue, sarcopenia, and mobility function in older individuals. J Aging Res. 2012;2012:629637. 22500231 10.1155/2012/629637 PMC3303569

[12] Tuttle LJ, Sinacore DR, Mueller MJ. Intermuscular adipose tissue is muscle specific and associated with poor functional performance. J Aging Res. 2012;2012:172957. 22666591 10.1155/2012/172957 PMC3361226

[13] Zacharias A, Pizzari T, English DJ, Kapakoulakis T, Green RA. Hip abductor muscle volume in hip osteoarthritis and matched controls. Osteoarthritis Cartilage. 2016;24(10):1727–35. 27163446 10.1016/j.joca.2016.05.002

[14] Fitzgerald G, Turiel G, Gorski T, Soro-Arnaiz I, Zhang J, Casartelli NC, et al. MME(+) fibro-adipogenic progenitors are the dominant adipogenic population during fatty infiltration in human skeletal muscle. Commun Biol. 2023;6(1):111. 36707617 10.1038/s42003-023-04504-y PMC9883500

[15] Naal FD, Sieverding M, Impellizzeri FM, von Knoch F, Mannion AF, Leunig M. Reliability and validity of the cross-culturally adapted German Oxford hip score. Clin Orthop Relat Res. 2009;467(4):952–7. 18726655 10.1007/s11999-008-0457-3 PMC2650060

[16] Naal FD, Impellizzeri FM, Leunig M. Which is the best activity rating scale for patients undergoing total joint arthroplasty? Clin Orthop Relat Res. 2009;467(4):958–65. 18587624 10.1007/s11999-008-0358-5 PMC2650053

[17] Casartelli NC, Lepers R, Maffiuletti NA. Assessment of the rate of force development scaling factor for the hip muscles. Muscle Nerve. 2014;50(6):932–8. 24585686 10.1002/mus.24229

[18] Casartelli NC, Maffiuletti NA, Item-Glatthorn JF, Staehli S, Bizzini M, Impellizzeri FM, et al. Hip muscle weakness in patients with symptomatic femoroacetabular impingement. Osteoarthritis Cartilage. 2011;19(7):816–21. 21515390 10.1016/j.joca.2011.04.001

[19] Lienhard K, Lauermann SP, Schneider D, Item-Glatthorn JF, Casartelli NC, Maffiuletti NA. Validity and reliability of isometric, isokinetic and isoinertial modalities for the assessment of quadriceps muscle strength in patients with total knee arthroplasty. J Electromyogr Kinesiol. 2013;23(6):1283–8. 24113423 10.1016/j.jelekin.2013.09.004

[20] Maffiuletti NA. Assessment of hip and knee muscle function in orthopaedic practice and research. J Bone Joint Surg Am. 2010;92(1):220–9. 20048117 10.2106/JBJS.I.00305

[21] Pfirrmann CW, Notzli HP, Dora C, Hodler J, Zanetti M. Abductor tendons and muscles assessed at MR imaging after total hip arthroplasty in asymptomatic and symptomatic patients. Radiology. 2005;235(3):969–76. 15860673 10.1148/radiol.2353040403

[22] Hermens HJ, Freriks B, Disselhorst-Klug C, Rau G. Development of recommendations for SEMG sensors and sensor placement procedures. J Electromyogr Kinesiol. 2000;10(5):361–74. 11018445 10.1016/s1050-6411(00)00027-4

[23] Missenard O, Mottet D, Perrey S. Factors responsible for force steadiness impairment with fatigue. Muscle Nerve. 2009;40(6):1019–32. 19623631 10.1002/mus.21331

[24] Wieser K, Joshy J, Filli L, Kriechling P, Sutter R, Fürnstahl P, et al. Changes of supraspinatus muscle volume and fat fraction after successful or failed arthroscopic rotator cuff repair. Am J Sports Med. 2019;47(13):3080–8. 31536372 10.1177/0363546519876289

[25] Sutter R, Kalberer F, Binkert CA, Graf N, Pfirrmann CW, Gutzeit A. Abductor tendon tears are associated with hypertrophy of the tensor fasciae latae muscle. Skelet Radiol. 2013;42(5):627–33. 10.1007/s00256-012-1514-2 22940837

[26] Takano Y, Kobayashi H, Yuri T, Yoshida S, Naito A, Kiyoshige Y. Fat infiltration in the gluteus minimus muscle in older adults. Clin Interv Aging. 2018;13:1011–7. 29872279 10.2147/CIA.S157402 PMC5973399

[27] Chi AS, Long SS, Zoga AC, Read PJ, Deely DM, Parker L, et al. Prevalence and pattern of gluteus medius and minimus tendon pathology and muscle atrophy in older individuals using MRI. Skelet Radiol. 2015;44(12):1727–33. 10.1007/s00256-015-2220-7 26260535

[28] Kivle K, Lindland E, Mjaaland KE, Pripp AH, Svenningsen S, Nordsletten L. The gluteal muscles in end-stage osteoarthritis of the hip: intra- and interobserver reliability and agreement of MRI assessments of muscle atrophy and fatty degeneration. Clin Radiol. 2018;73(7):675.e17-.e24. 29587967 10.1016/j.crad.2018.02.014

[29] Muller M, Tohtz S, Winkler T, Dewey M, Springer I, Perka C. MRI findings of gluteus minimus muscle damage in primary total hip arthroplasty and the influence on clinical outcome. Arch Orthop Trauma Surg. 2010;130(7):927–35. 20221834 10.1007/s00402-010-1085-4

[30] Woodley SJ, Nicholson HD, Livingstone V, Doyle TC, Meikle GR, Macintosh JE, et al. Lateral hip pain: findings from magnetic resonance imaging and clinical examination. J Orthop Sports Phys Ther. 2008;38(6):313–28. 18515960 10.2519/jospt.2008.2685

[31] Nozaki T, Tasaki A, Horiuchi S, Ochi J, Starkey J, Hara T, et al. Predicting retear after repair of full-thickness rotator cuff tear: two-point dixon MR imaging quantification of fatty muscle degeneration-initial experience with 1-year follow-up. Radiology. 2016;280(2):500–9. 26937710 10.1148/radiol.2016151789

[32] Collins KH, Hart DA, Reimer RA, Seerattan RA, Waters-Banker C, Sibole SC, et al. High-fat high-sucrose diet leads to dynamic structural and inflammatory alterations in the rat vastus lateralis muscle. J Orthop Res. 2016;34(12):2069–78. 26990324 10.1002/jor.23230

[33] Blocquiaux S, Gorski T, Van Roie E, Ramaekers M, Van Thienen R, Nielens H, et al. The effect of resistance training, detraining and retraining on muscle strength and power, myofibre size, satellite cells and myonuclei in older men. Exp Gerontol. 2020;133:110860. 32017951 10.1016/j.exger.2020.110860

[34] Blocquiaux S, Gorski T, Van Roie E, Ramaekers M, Van Thienen R, Nielens H, et al. Corrigendum to “The effect of resistance training, detraining and retraining on muscle strength and power, myofibre size, satellite cells and myonuclei in older men” [Exp. Gerontol., 133, 2020, 110860]. Exp Gerontol. 2020;134:110897. 32147251 10.1016/j.exger.2020.110897

[35] Nederveen JP, Joanisse S, Snijders T, Ivankovic V, Baker SK, Phillips SM, et al. Skeletal muscle satellite cells are located at a closer proximity to capillaries in healthy young compared with older men. J Cachexia Sarcopenia Muscle. 2016;7(5):547–54. 27239425 10.1002/jcsm.12105 PMC4864218

[36] Schneider CA, Rasband WS, Eliceiri KW. NIH Image to ImageJ: 25 years of image analysis. Nat Methods. 2012;9(7):671–5. 22930834 10.1038/nmeth.2089 PMC5554542

[37] Wen Y, Murach KA, Vechetti IJ Jr, Fry CS, Vickery C, Peterson CA, et al. MyoVision: software for automated high-content analysis of skeletal muscle immunohistochemistry. J Appl Physiol (1985). 2018;124(1):40–51. 28982947 10.1152/japplphysiol.00762.2017 PMC6048460

[38] Kelly NA, Hammond KG, Stec MJ, Bickel CS, Windham ST, Tuggle SC, et al. Quantification and characterization of grouped type I myofibers in human aging. Muscle Nerve. 2018;57(1):E52-e9. 28561923 10.1002/mus.25711 PMC5711619

[39] Hepple RT, Mackinnon SL, Thomas SG, Goodman JM, Plyley MJ. Quantitating the capillary supply and the response to resistance training in older men. Pflugers Arch. 1997;433(3):238–44. 9064638 10.1007/s004240050273

[40] Impellizzeri FM, Mannion AF, Naal FD, Leunig M. A Core Outcome Measures Index (COMI) for patients undergoing hip arthroplasty. J Arthroplasty. 2013;28(9):1681–6. 23523492 10.1016/j.arth.2013.01.014

[41] Julious S. Sample size of 12 per group rue of thumb for a pilot study. Pharm Stat. 2005;4:287–91.

[42] West RM. Best practice in statistics: the use of log transformation. Ann Clin Biochem. 2022;59(3):162–5. 34666549 10.1177/00045632211050531 PMC9036143

[43] Loureiro A, Mills PM, Barrett RS. Muscle weakness in hip osteoarthritis: a systematic review. Arthritis Care Res (Hoboken). 2013;65(3):340–52. 22833493 10.1002/acr.21806

[44] Suetta C, Aagaard P, Magnusson SP, Andersen LL, Sipila S, Rosted A, et al. Muscle size, neuromuscular activation, and rapid force characteristics in elderly men and women: effects of unilateral long-term disuse due to hip-osteoarthritis. J Appl Physiol (1985). 2007;102(3):942–8. 17122381 10.1152/japplphysiol.00067.2006

[45] Perraton Z, Mosler AB, Lawrenson PR, Weber Ii K, Elliott JM, Wesselink EO, et al. The association between lateral hip muscle size/intramuscular fat infiltration and hip strength in active young adults with long standing hip/groin pain. Phys Ther Sport. 2024;65:95–101. 38101293 10.1016/j.ptsp.2023.11.007

[46] Willemse H, Theodoratos A, Smith PN, Dulhunty AF. Unexpected dependence of RyR1 splice variant expression in human lower limb muscles on fiber-type composition. Pflugers Arch. 2016;468(2):269–78. 26438192 10.1007/s00424-015-1738-9

[47] Sĭrca A, Susec-Michieli M. Selective type II fibre muscular atrophy in patients with osteoarthritis of the hip. J Neurol Sci. 1980;44(2–3):149–59. 6444440 10.1016/0022-510x(80)90123-9

[48] Verdijk LB, Snijders T, Drost M, Delhaas T, Kadi F, van Loon LJ. Satellite cells in human skeletal muscle; from birth to old age. Age (Dordr). 2014;36(2):545–7. 24122288 10.1007/s11357-013-9583-2 PMC4039250

[49] Fink B, Egl M, Singer J, Fuerst M, Bubenheim M, Neuen-Jacob E. Morphologic changes in the vastus medialis muscle in patients with osteoarthritis of the knee. Arthritis Rheum. 2007;56(11):3626–33. 17968889 10.1002/art.22960

[50] Lexell J, Downham DY. The occurrence of fibre-type grouping in healthy human muscle: a quantitative study of cross-sections of whole vastus lateralis from men between 15 and 83 years. Acta Neuropathol. 1991;81(4):377–81. 2028741 10.1007/BF00293457

[51] Nakamura T, Suzuki K. Muscular changes in osteoarthritis of the hip and knee. Nihon Seikeigeka Gakkai Zasshi. 1992;66(5):467–75. 1387155

[52] Dulor JP, Cambon B, Vigneron P, Reyne Y, Nouguès J, Casteilla L, et al. Expression of specific white adipose tissue genes in denervation-induced skeletal muscle fatty degeneration. FEBS Lett. 1998;439(1–2):89–92. 9849884 10.1016/s0014-5793(98)01216-2

[53] Uezumi A, Fukada S, Yamamoto N, Takeda S, Tsuchida K. Mesenchymal progenitors distinct from satellite cells contribute to ectopic fat cell formation in skeletal muscle. Nat Cell Biol. 2010;12(2):143–52. 20081842 10.1038/ncb2014

[54] Kopinke D, Roberson EC, Reiter JF. Ciliary hedgehog signaling restricts injury-induced adipogenesis. Cell. 2017;170(2):340-51.e12. 28709001 10.1016/j.cell.2017.06.035 PMC5617351

[55] Noehren B, Kosmac K, Walton RG, Murach KA, Lyles MF, Loeser RF, et al. Alterations in quadriceps muscle cellular and molecular properties in adults with moderate knee osteoarthritis. Osteoarthritis Cartilage. 2018;26(10):1359–68. 29800621 10.1016/j.joca.2018.05.011 PMC7050996

[56] Sambasivan R, Yao R, Kissenpfennig A, Van Wittenberghe L, Paldi A, Gayraud-Morel B, et al. Pax7-expressing satellite cells are indispensable for adult skeletal muscle regeneration. Development. 2011;138(17):3647–56. 21828093 10.1242/dev.067587

