# Supplementary figures and images for: Intramuscular fatty infiltration and its correlation with muscle composition and function in hip osteoarthritis
Source: Skelet Muscle. 2024 Dec 19;14:32. doi: 10.1186/s13395-024-00364-0 (PMC11657216; doi:10.1186/s13395-024-00364-0)

## Flow Diagram

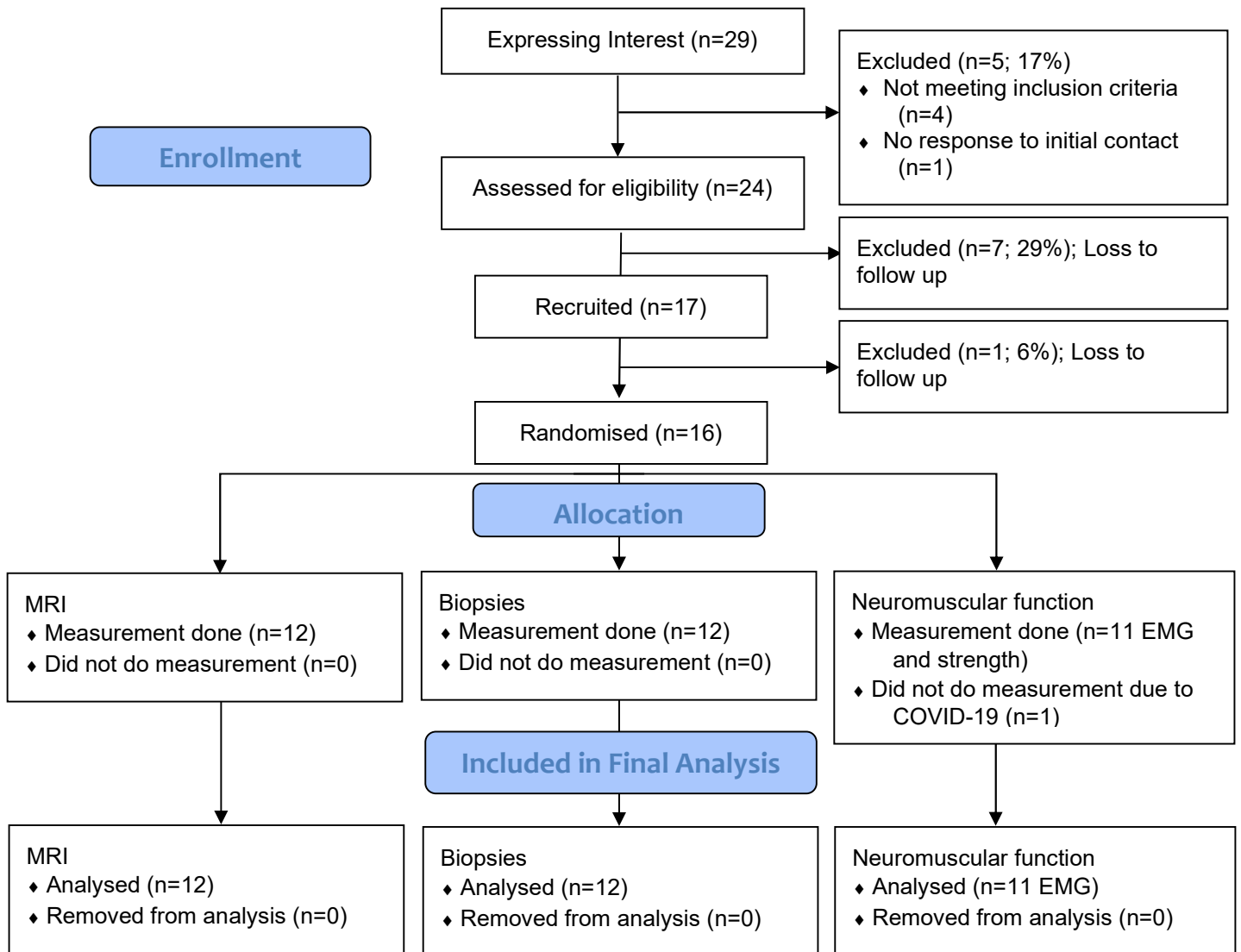

Supplement: Supplementary file 1 — Supplementary Material 1: Suppl. Fig. 1: Flow chart. [file 13395_2024_364_MOESM1_ESM.pdf]

Study outline

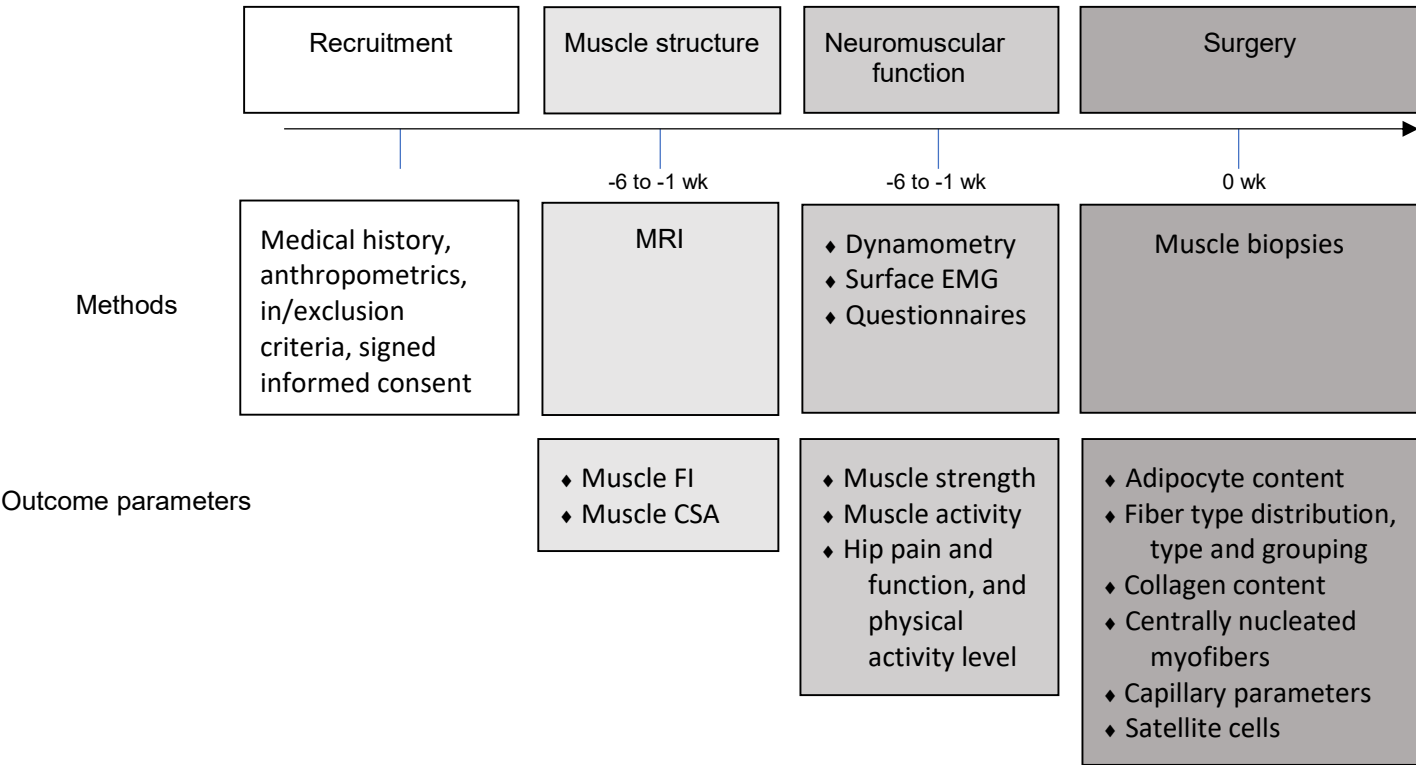

Supplement: Supplementary file 2 — Supplementary Material 2: Suppl. Fig. 2: Study outline. [file 13395_2024_364_MOESM2_ESM.pdf]
